# Comment on Tyson, G.; Wild, J. Post-Traumatic Stress Disorder Symptoms among Journalists Repeatedly Covering COVID-19 News. *Int. J. Environ. Res. Public Health* 2021, *18*, 8536

**DOI:** 10.3390/ijerph182111421

**Published:** 2021-10-30

**Authors:** Roel Van Overmeire

**Affiliations:** Mental Health and Wellbeing Research Group, Department of Public Health, Vrije Universiteit Brussel, 1090 Brussel, Belgium; roel.van.overmeire@vub.be

In the article “Post-Traumatic Stress Disorder Symptoms among Journalists Repeatedly Covering COVID-19 News” by Tyson & Wild [1], the authors investigated the mental health, consisting of general mental health, depression and post-traumatic stress disorder (PTSD), among journalists during COVID-19. Although it is interesting in its scope and goals, I believe the article has several issues in its methodology and interpretation.

The first issue is the use of PTSD. In the DSM-V, PTSD can only occur among people who fit the inclusion conditions of criterion A. This includes exposure to events where someone threatened death, serious injury or sexual violence [2]. Thus, before the PCL-5 (the scale to assess PTSD in this study) is completed, it needs to be determined that all people fit criterion A; thus, whether they have actually experienced trauma. The authors did indeed measure trauma, but never made it clear that all the participants had experienced trauma. In fact, we receive little information on the trauma outcomes. Thus, if there were participants who had not experienced any trauma at all, then they should have been excluded from completing the PCL-5. Furthermore, the PCL-5 should generally be completed with regards to a specific traumatic event–in this case, it is not clear which one. The text seems to insinuate that the PCL-5 can also be related to COVID-19 as trauma, considering that the authors argued that COVID-19 has a severe impact on the mental health on journalists. 

However, if the authors would argue that COVID-19 is a trauma, then it becomes dubious. The sample consists of journalists in all forms: reporters, editors, camera operators etc. Yet, already from this description we might conclude that most of these would not fit criterion A of PTSD. Journalists such as editors are not people who directly experience such events. In fact, editors are the type of people that experience events in a way that is explicitly excluded from a diagnosis for PTSD: inclusion through media-watching of a trauma [2]. Are the camera operators the ones filming the coverage, or filming the news broadcasters? Et cetera. 

Furthermore, the case can be made that simply *reporting* on COVID-19 would not fit criterion A. Considering that PTSD consists of a fight-or-flight response, where would it originate in the reporting of COVID-19? Several authors have already shown that in essence, if we follow the DSM-V description, COVID-19 is not a trauma and thus not fitting for PTSD [3,4]. In fact, many of the elements that the authors mention, namely working late hours, experiencing a serious disease, and being stressed, are all inclusions for adjustment disorders as defined in the ICD-11 [5]. In fact, scientifically speaking, it would be more interesting to study adjustment disorders, as they are largely neglected by the scientific community, despite them being diagnosed regularly by clinicians [6,7,8]. 

The widening of the inclusions for PTSD are typical for a phenomenon where scientists widen definitions to fit their own research [9,10]. This is problematic, as it leads to the overestimation of disorders, because, simply put: if you widen your inclusion criteria then, obviously, more people will fit the description [11]. This allowed for the somewhat strange comparison of the study sample with a sample of war-journalists, where the authors show that their sample has a higher percentage of probable PTSD, when compared to journalists who have been in war situations. Thus, COVID-19-journalists would have a higher percentage of PTSD, which seems somewhat counterintuitive. 

A typical facet of such a widening of inclusion criteria is that authors will start using terms referring to people who half-fit the PTSD diagnosis, without fully fitting the diagnosis [11]. The problem of creating a category for people who do not fit the full diagnosis of PTSD is that it is a way of problematizing possibly normal behavior. Furthermore, it is arbitrary, and it is often not based on actual psychological processes. In this article, the authors used “sub-threshold PTSD”. This term was used to refer to people that have a high impairment due to PTSD symptoms, without having PTSD. Yet, the problem of using this term is that, firstly, it has no basis in the DSM-V, and secondly, the authors based themselves on research from 1997 by Stein et al. [12]. However, for Stein et al. [12], their research was based on the DSM-IV, not the DSM-V. Furthermore, they called their form of sub-threshold PTSD “partial PTSD”, which already indicated that the authors of the current study made their own form of “almost-PTSD”. This is confirmed by the fact that the authors used other criteria for their sub-threshold PTSD category when compared to Stein et al. [12], making it strange that they even referred to the 1997 study for their own PTSD variation. Variations such as partial PTSD or sub-threshold PTSD remain quite controversial, precisely because it creates disorders where there is no agreement that there is a disorder [13]. If the argument is that “sub-threshold” PTSD causes serious impairment, then where do we draw the line? Why then not report on all symptom criteria individually? Why would having only criterion D symptoms (such as detachment) be less worthy to report on? This too would impair the social functioning of a person. We do not report on it, because it does not fit what is agreed upon on in the guides we base our diagnoses or screenings on, namely the DSM-V or the ICD-11. That does not mean that these people had no issues. It does, however, mean that we do not see their problems as a mental disorder. 

In the Results section, I found little on the trauma screening itself. However, the authors did state that the lifetime trauma prevalence was significantly different between the group that reported on COVID-19 and the group that did not report on COVID-19. This is problematic because, firstly, it might indicate that not all the participants had experienced a trauma, meaning that not everyone should have completed the PCL-5, and secondly, that all the conclusions that the authors drew that were based on the PCL-5 were already biased. If one group has less trauma exposure, then typically their PCL-5 will also be lower. Thus, for example, if a person has had many trauma experiences (for example, due reporting on war), then to report during COVID-19 and have a higher PCL-5 value does not mean anything. In fact, it just means that experiencing trauma can lead to PTSD, but COVID-19 has nothing to do with it in this case. 

In general, it remains unclear what COVID-19 has to do with the PCL-5 scores. Because on the one hand, the authors measured lifetime trauma (which would be correct for the inclusion for the PCL-5). On the other hand, they compared the PCL-5 for two groups on the basis of COVID-19 coverage. As COVID-19 is not a trauma, it becomes unclear why this was done. What can be learned from the fact that, for example, a person experienced a life-threatening situation 5 years ago, has problems due to this and now reports on COVID-19? This does not say anything about COVID-19 and its “impact” on people. Else, one might assume that if people had mental health problems during non-COVID-19 times, this was due to the reporting of global climate change, or due to reporting on crime, or anything, really. Associations can be made between any variables–however, that does not mean it always makes sense to do so. 

COVID-19 has given rise to an oversimplification of PTSD. This is unfortunate, as guides such as the DSM-V are ideal to unify research on PTSD, if researchers follow the guide. However, as the conditions are widened, research becomes impossible to compare, thus halting our progress in terms of understanding PTSD. 

The second issue with the paper is in the statistics. Firstly, I wonder if the authors checked their assumptions before starting their linear models. For example, when you look at the PCL mean score, we see that the standard deviation is almost as large as the mean, which is often a good indication of failing some assumptions. Secondly, one could argue that the linear regression also suffers from a form of self-confirmation. For example, “numbing” can be considered part of PTSD–if you look at criterion D of PTSD, it seems related to the inability to experience positive emotions. Thus, if we predict PTSD symptoms through another PTSD symptom, then of course we get a *p*-value lower than 0.05, because people who have PTSD will have symptoms of numbing. The same goes for “thought suppression”–in fact, one might argue that this is part of criterion C of PTSD [2]. In fact, rumination is at least shown to be strongly associated with PTSD [14]. It is therefore no surprise that in this regression, despite the small sample, 53% of the variation was explained, because PTSD is predicted on the basis of PTSD symptoms. 

In relation to this, I wonder if the authors checked for multicollinearity between the variables. Is “numbing” radically different from “thought suppression” in terms of association? If nothing else, adding so many variables into one model would likely cause the predictors to correlate strongly with each other. For example, to predict someone’s income, it is generally not recommended statistically to predict it with someone’s current capital, or with their job position, because all these things tend to correlate strongly with each other. 

There is also a contradiction in the paper which I do not understand: in Table 1, we see that there are two groups. Group one is the group of people who covered COVID-19, and the other group is the group that did not cover COVID-19. However, then we see that there is a variable called “Interviewing people with COVID-19”. Firstly, logic would dictate that in the group of people that did not report on COVID-19, there should be zero people interviewing people with COVID-19. Thus, there is already an enormous bias towards finding a significant *p*-value, because obviously there is going to be a difference between the groups if the variables literally only apply to one group. Secondly, in contradiction to the point of these groups, we see that six people who did not report on COVID-19 had interviewed people with COVID-19. How is it that someone can interview a person with COVID-19, but not do a story on COVID-19?

The third issue with the paper concerns the interpretation. In the conclusion, the authors stated that people reporting on COVID-19 had more psychological distress. However, for both groups, the mean values for the GHQ-12 (measuring psychological distress) were very high. They were both close to the maximum score of 36 (M = 29.7 and M = 27.09). Simply stating that there is a significant *p*-value ignores the fact that both the means for both groups were very high. The conclusion that should have been made on the basis of the GHQ-12 values is that journalists in general have a lot of distress problems. 

The authors also discuss the “impact” of PTSD on journalists. It is unclear how a disorder can have an impact (one would expect that reporting on COVID-19 has an impact on PTSD development). Another problem is that the authors draw causal conclusions based on a cross-sectional study. It is impossible to draw such conclusions. Thus, statements such as “We were interested in documenting the effects of repeated reporting of COVID-19 on the mental health of journalists” were impossible to investigate with the study design, as well as how mental health was measured (as seen in the first issue I discussed). 

It is also surprising that the authors did not report on their low depression values. PTSD is, generally, moderately correlated with depression, thus this finding is interesting. Furthermore, the discrepancy between the GHQ-12 scores and the PHQ-9 scores was quite large, considering that the GHQ-12 has some questions that measure depression. The COVID-19 reporters had a mean of 7.86 which would roughly translate to moderate depressive symptoms, which is not ideal, of course, but much lower than might be expected based on the GHQ-12 score. In fact, it is very surprising, since the GHQ-12 also includes depression symptoms. As a comparison, the PHQ-9 ranged from 0 to 27, where the COVID-19 reporters had a mean of 7.86, while the GHQ-12 ranged from 0 to 36, where the COVID-19 reporters had a mean score of 29.7. Thus, it is unfortunate that the authors did not report that despite an enormously high GHQ-12 score, there was no positive screening for depression, globally speaking, nor was there a positive screening for PTSD, globally speaking. This would indicate that other mental health issues trouble journalists. In fact, I would conclude that neither PTSD nor depression is the problem, but other mental health issues. Again, the GHQ-12 was incredibly high in this study. It is unfortunate that the authors did not include on which criteria the respondents scored high on the PCL-5 (as the symptom clusters are part of different symptom criteria of PTSD in the DSM-V), as it might have given us a clue as to what factors were troublesome for the journalists. It is clear that it had hardly anything to do with covering COVID-19, as the difference between the group covering COVID-19 and the group not covering COVID-19 was 2.61 points in the mean scores (29.7 and 27.09). 

Thus, to summarize: firstly, there is unclarity on what basis people completed the PCL-5; was everyone included for the PCL-5 based on their trauma exposure? Secondly, considering this was a lifetime trauma screening, how can the authors be certain that COVID-19 had anything to do with the symptoms? Thirdly, the statistics in the paper are somewhat unclear. Finally, the interpretation of some of the results were, in my view, not exact. 
